# Chronic kidney disease in Russia: the Ural eye and medical study

**DOI:** 10.1186/s12882-020-01843-4

**Published:** 2020-05-25

**Authors:** Mukharram M. Bikbov, Rinat M. Zainullin, Gyulli M. Kazakbaeva, Timur R. Gilmanshin, Ellina M. Rakhimova, Iulia A. Rusakova, Natalia I. Bolshakova, Kamila R. Safiullina, Songhomitra Panda-Jonas, Dilya F. Yakupova, Nikolai A. Nikitin, Jost B. Jonas

**Affiliations:** 1 Ufa Eye Research Institute, Ufa, Bashkortostan Russia; 2 Department of Ophthalmology, Medical Faculty Mannheim, Heidelberg University, Theodor-Kutzer-Ufer 1, 68167 Mannheim, Germany

**Keywords:** Arterial hypertension, Chronic kidney disease, Diabetes mellitus, Glomerular filtration rate

## Abstract

**Background:**

To examine the prevalence of chronic kidney disease (CKD) and its associations in a Russian population.

**Methods:**

Out of 7328 eligible individuals, the population-based cross-sectional Ural Eye and Medical Study included 5899 (80.5%) individuals aged 40+ years and undergoing a detailed medical examination. The estimated glomerular filtration rate (eGFR) was calculated using the Chronic-Kidney-Disease-Epidemiology-Collaboration (CKD-EPI) equation.

**Results:**

The mean eGFR was 72.3 ± 19.1 mL/min/1.73m^2^ (median:70.3 mL/min/1.73m^2^). Prevalence of CKD stage 3a (eGFR< 60 mL/min/1.73m^2^ and > 45 mL/min/1.73m^2^), CKD stage 3b (eGFR< 45 mL/min/1.73m^2^ and > 30 mL/min/1.73m^2^) and CKD stage 4+ (eGFR< 30 mL/min/1.73m^2^) were 1351/5841 (23.1%;95% confidence interval (CI):22.1,24.2), 294/5841 (5.0%;95%CI:4.5,5.6), and 29/5841 (0.5%;95%CI:0.3,0.7), respectively. The CKD stage 3+ prevalence increased (*P* < 0.001) from 11.1% (95%CI:8.4,13.9) in 40–44-year-olds to 56.8% (95%CI:52.8,60.8) in 75 + year-olds. In univariate analysis, CKD stage 3a + prevalence increased with higher systolic blood pressure (*P* < 0.001). In multivariable analysis, higher prevalence of CKD stage 3a + was associated with older age (*P* < 0.001;odds ratio (OR):1.06;95%CI:1.05,1.07), female sex (*P* < 0.001;OR:2.29;95%CI:1.94,2.69), rural region of habitation (*P* = 0.001;OR:1.29;95%CI:1.11,1.50), higher body mass index (*P* = 0.03;OR:1.02;95%CI:1.002,1.03), lower prevalence of house ownership (*P* = 0.02;OR:0.57;95%CI:0.35,0.92), higher prevalence of mostly sitting or standing during work (*P* < 0.001;OR:1.40;95%CI:1.20,1.64), higher serum concentration of triglycerides (*P* < 0.001;OR:1.23;95%CI:1.12,1.35) and blood urea nitrogen (*P* < 0.001;OR:1.33;95%CI:1.27,1.40), lower serum concentration of hemoglobin (*P* = 0.03;OR:0.99;95%CI:0.99,0.999), and lower prevalence of chronic obstructive pulmonary disease (*P* < 0.001;OR:0.57;95%CI:0.42,0.78).

**Conclusions:**

In this population from Russia aged 40+ years, prevalence of CKD stage 3+ (28.7%;95%CI:27.5,29.8) was relatively high as compared to populations from other countries. Associated factors were older age, female sex, rural region, higher body mass index, a sedentary lifestyle, and lower socioeconomic background.

## Background

The recent Global Burden of Diseases, Injuries, and Risk Factors Study GBD 2017 revealed that chronic kidney disease (CKD) caused 1.23 million deaths (95% confidence interval (CI): 1.20, 1.26) worldwide in 2017 and that this figure had increased by 34.2% (95%CI: 32.0, 36.2) in the preceding decade [1]. CKD was the 16th most common reason of global YLLs (Years of Live Lost) in 2017, and it caused 35.8 million DALYs (disability-adjusted life years) (95%CI: 33.7, 38.0). Its age-standardized death rate per 100,000 was 33.6 (95%CI: 32.9, 34.4) [1]. In addition, an impaired kidney function (defined as a glomerular filtration rate (GFR) of < 60 mL/min/1.73m^2^ or by an albumin-to-creatinine ratio > 30 mg/g, after exclusion of end-stage renal disease), caused 2.59 million deaths (95%CI: 2.39, 2.80) in 2017 and 61.3 million DALYS (95%CI: 56.9, 66.1) [2]. Despite the high public health importance of a low GFR, information about the prevalence of CKD in Russia has been scarce so far [3–10]. We therefore conducted this study to assess the frequency of CKD in a population in Russia and explored associations of CKD with other systemic factors such as gender, age, region of habitation, socioeconomic parameters, and medical factors such as biochemical blood parameters and arterial blood pressure.

## Methods

The Ural Eye and Medical Study (UEMS) is a population-based study performed in the urban region of Kirovskii of the city of Ufa and in villages of the rural region of the Karmaskalinsky District in a distance of 65 km from Ufa. Ufa is the capital of the republic of Bashkortostan and has a population of 1.1 million inhabitants including Russians, Bashkirs, Tatars, Ukrainians and other ethnicities. It is located at the south-western end of the Ural Mountains. The Ethics Committee of the Academic Council of the Ufa Eye Research Institute approved the study design, with informed written consent obtained from all participants. The study period ranged from 2015 to 2017 [11–13]. Inclusion criteria were living in the study regions and an age of 40 years or older. Out of all (*n* = 7328) individuals living in the study region and having an age of 40 + years, 5899 (80.5%) individuals participated in the study. Social workers visited all eligible study participants at home and invited them to come to the hospital for the examination. If the individuals were not met in their homes or hesitated to participate in the study, the social workers returned up to three times to the homes of the individuals.

All study participants underwent a series of examinations including an interview (conducted by trained social workers and consisting of more than 250 standardized questions on the socioeconomic background, smoking habits and alcohol consumption, physical activity, depression and anxiety, and known diagnosis and therapy of major diseases), anthropometry, blood pressure measurement, handgrip dynamometry, spirometry, and biochemical analysis of blood samples taken under fasting conditions. Using a BS3000P analyzer, we applied Jaffe’s kinetic method for the determination of the serum creatinine concentration without de-proteinization. As part of the clinical routine of the university hospital-associated clinical laboratory, the diagnostic accuracy of the serum concentration measurements was regularly checked in 6-months intervals. The Guidelines for Accurate and Transparent Health Estimates Reporting (GATHER statement guidelines) were applied for collecting the data [14]. Applying the new guidelines of the American College of Cardiology and the American Heart Association for the detection, prevention, management and treatment of high blood pressure (BP), we differentiated between normal BP (systolic BP (SBP) / diastolic BP (DBP) less than 120/80 mmHg), elevated BP (SBP between 120 and 129 mmHg and DBP less than 80 mmHg), stage 1 of hypertension (SBP between 130 and 139 mmHg or DBP between 80 and 89 mmHg), stage 2 of hypertension (SBP at least 140 mmHg and ≤ 180 mmHg or DBP at least 90 mmHg and ≤ 120 mmHg), and a hypertensive crisis (SBP > 180 mmHg and/or DBP > 120) [15, 16]. Criteria for diabetes mellitus were a fasting serum glucose concentration of ≥7.0 mmol/L or a self-reported history of physician-based diagnosis or therapy of diabetes mellitus. The study design has been described in detail recently [11–13].

We calculated the estimated glomerular filtration rate (eGFR) using the CKD Epidemiology Collaboration (CKD-EPI) equation [17–20]. Stage 3a of CKD was defined as an eGFR of < 60 mL/min/1.73 m^2^ and ≥ 45 mL/min/1.73 m^2^, Stage 3b as an eGFR of < 45 mL/min/1.73 m^2^ and ≥ 30 mL/min/1.73 m^2^, stage 4 as an eGFR of < 30 mL/min/1.73 m^2^ and ≥ 15 mL/min/1.73 m^2^, and stage 5 as an eGFR of < 15 mL/min/1.73 m^2^. Since we did not assess albuminuria/proteinuria in our study, CKD stages 1 and 2 could not be defined.

The data were statistically analyzed using a statistical software package (SPSS for Windows, version 25.0, IBM-SPSS, Chicago, IL, USA). We first assessed the mean values of the main outcome parameter, i.e. GFR, (presented as mean ± standard deviation) and the prevalence of CKD (presented as frequency and 95% confidence intervals (CI)). We then carried out a logistic regression analysis of associations between the prevalence of CKD stage 3a + as dependent variable and other systemic variables, after adjusting for age. It was followed by a multivariable logistic regression analysis, in which the prevalence of CKD stage 3a + was the dependent variable and independent variables were all those parameters which were associated (*P* < 0.10) with the CKD stage 3a + prevalence in the previous analysis. We compared the eGFR between the various arterial hypertension stages by a one way ANOVA (analysis of variance). We assessed the relationship between systolic blood pressure and eGFR by linear regression analysis. We calculated the odds ratios (OR) and their 95% CIs. All *P*-values were two-sided and considered statistically significant, if the values were less than 0.05.

## Results

Measurements of the blood concentration of creatinine were available for 5841 (99.0%) individuals out of the 5899 individuals who primarily participated in the Ural Eye and Medical Study. The group of subjects with information on creatinine and the group of individuals without creatinine measurements did not differ significantly in age (58.9 ± 10.7 years versus 61.9 ± 12.0 years; *P* = 0.06), gender (men/women: 43.7%/56.3% versus 52%/48%; *P* = 0.23) and level of education (*P* = 0.92). The distribution of age and gender did not vary markedly between the study population and the whole population of Russia (as assessed in the most recent census performed in 2010), with two constrictions in both populations due to the consequences of World War II. The mean age of the study population was 58.9 ± 10.7 years (median: 58 years; range: 40–94 years) [21].

**Fig. 1:**
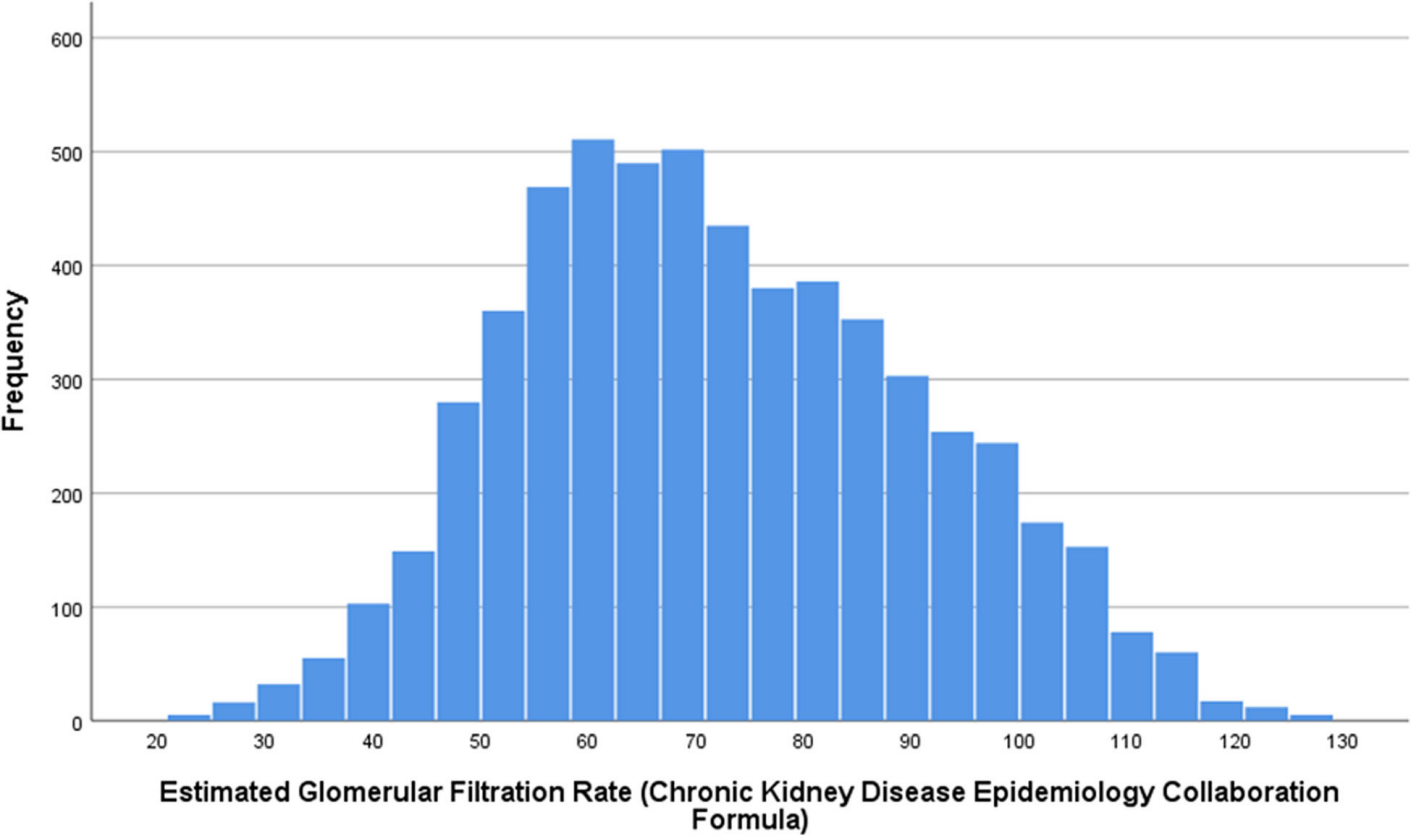
Histogram showing the distribution of the estimated glomerular filtration rate in the Ural Eye and Medical Study

The mean creatinine concentration was 89.9 ± 25.0 μmol/L (median: 88.5 μmol/L) and the mean eGFR was 72.3 ± 19.1 mL/min/1.73m^2^ (median: 70.3 mL/min/1.73m^2^) (Fig. 1). Out of the 5841 study participants, 1351 (23.1% (95%CI: 22.1, 24.2)) individuals had a CKD stage 3a, 294 (5.0% (95%CI: 4.5, 5.6)) individuals had a CKD stage 3b, and 29 individuals had a CKD stage 4 or 5 (0.5% (95%CI: 0.3, 0.7)). There were three individuals (3/5841; 0.05%) with a CKD stage 5. The prevalence of CKD stage 3+ was 1674/5841 or 28.7% (95%CI: 27.5, 29.8).

**Fig. 2:**
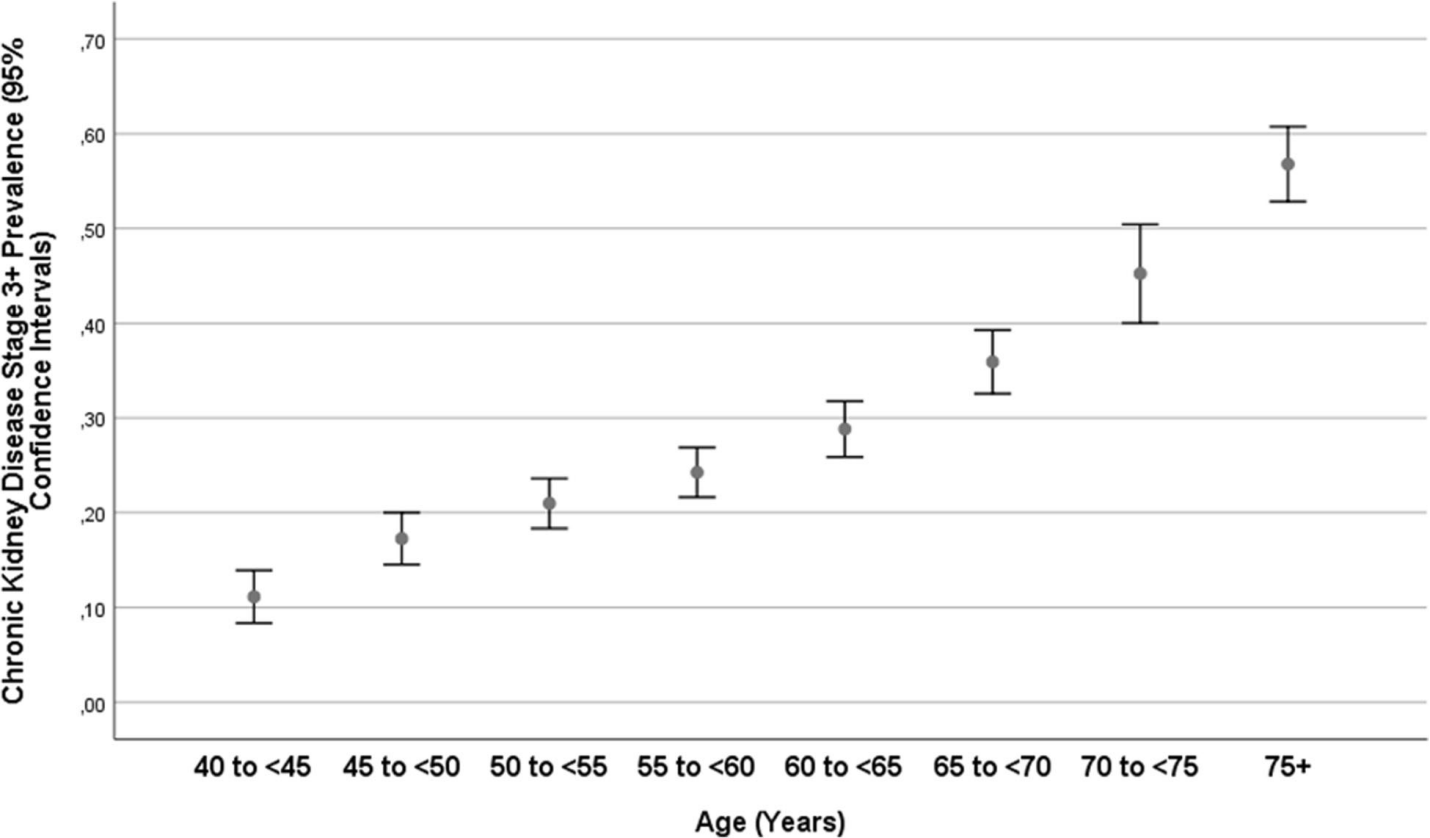
Graph showing the distribution of the prevalence of chronic kidney disease stage 3+ (estimated glomerular filtration rate < 60 mL/min/1.73m^2^) stratified by age in the Ural Eye and Medical Study

**Fig. 3:**
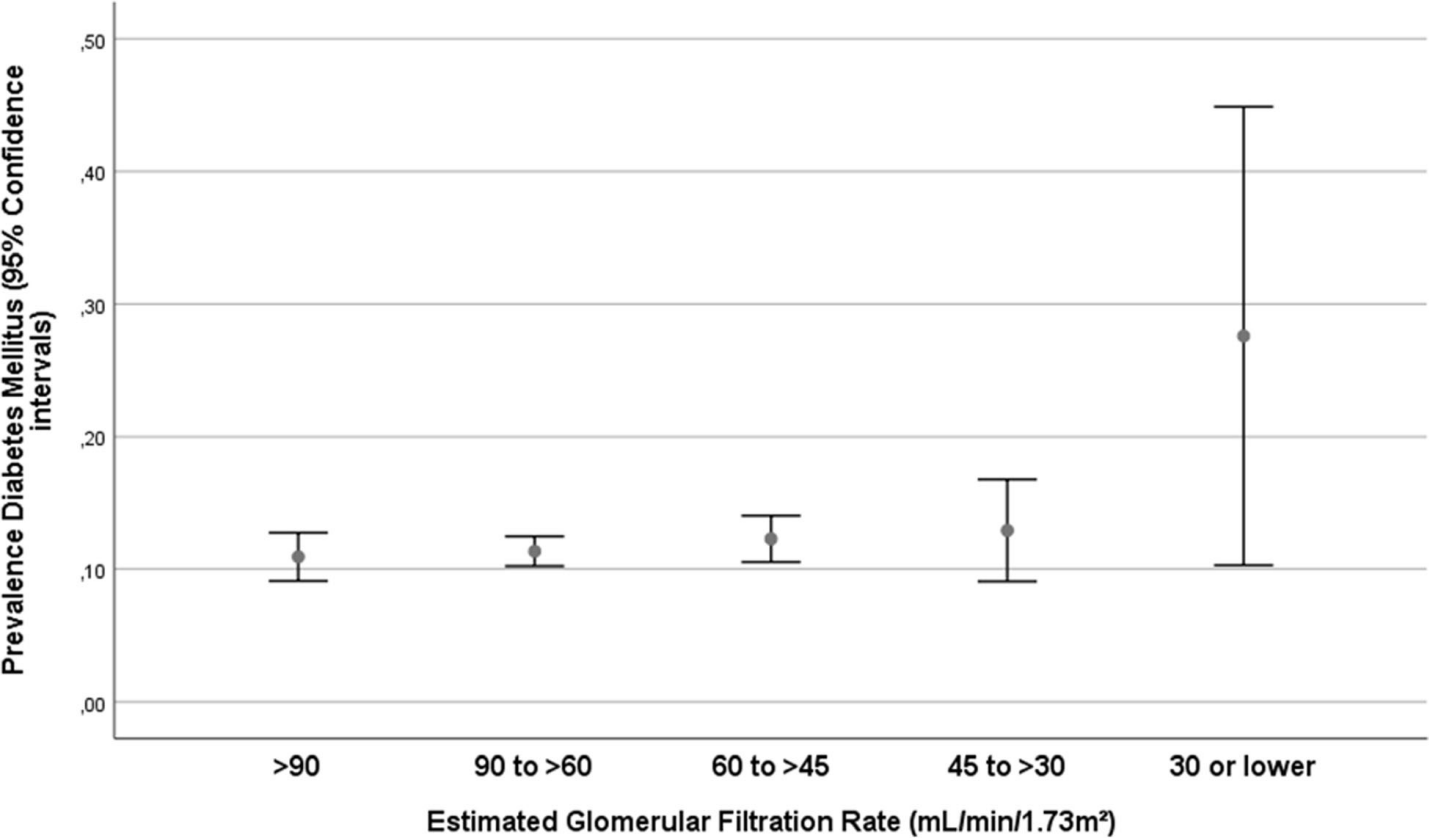
Graph showing the distribution of the prevalence of diabetes mellitus stratified by the chronic kidney disease stage in the Ural Eye and Medical Study

The group of participants with a CKD stage 3a or higher (i.e., eGFR < 60 mL/min/1.73m^2^) differed from the remaining individuals in older age (63.6 ± 11.0 years versus 57.1 ± 9.9 years; *P* < 0.001) (Fig. 2), and had a higher, but not significantly higher, prevalence of diabetes mellitus (12.7% (95%CI: 11.1, 14.3) versus 11.2% (95%CI: 10.3, 12.2); *P* = 0.13) (Fig. 3). The CKD stage 3+ prevalence increased from 11.1% (95%CI: 8.4, 13.9) in the age group from 40 to 44 years, to 21.0% (95%CI: 18.3, 23.6) in the age group from 50 to 54 years, to 28.8% (95%CI: 25.9, 31.8) in the age group from 60 to 64 years, and to 56.8% (95%CI: 52.8, 60.8) in the age group of 75+ years (Fig. 2).

**Fig. 4:**
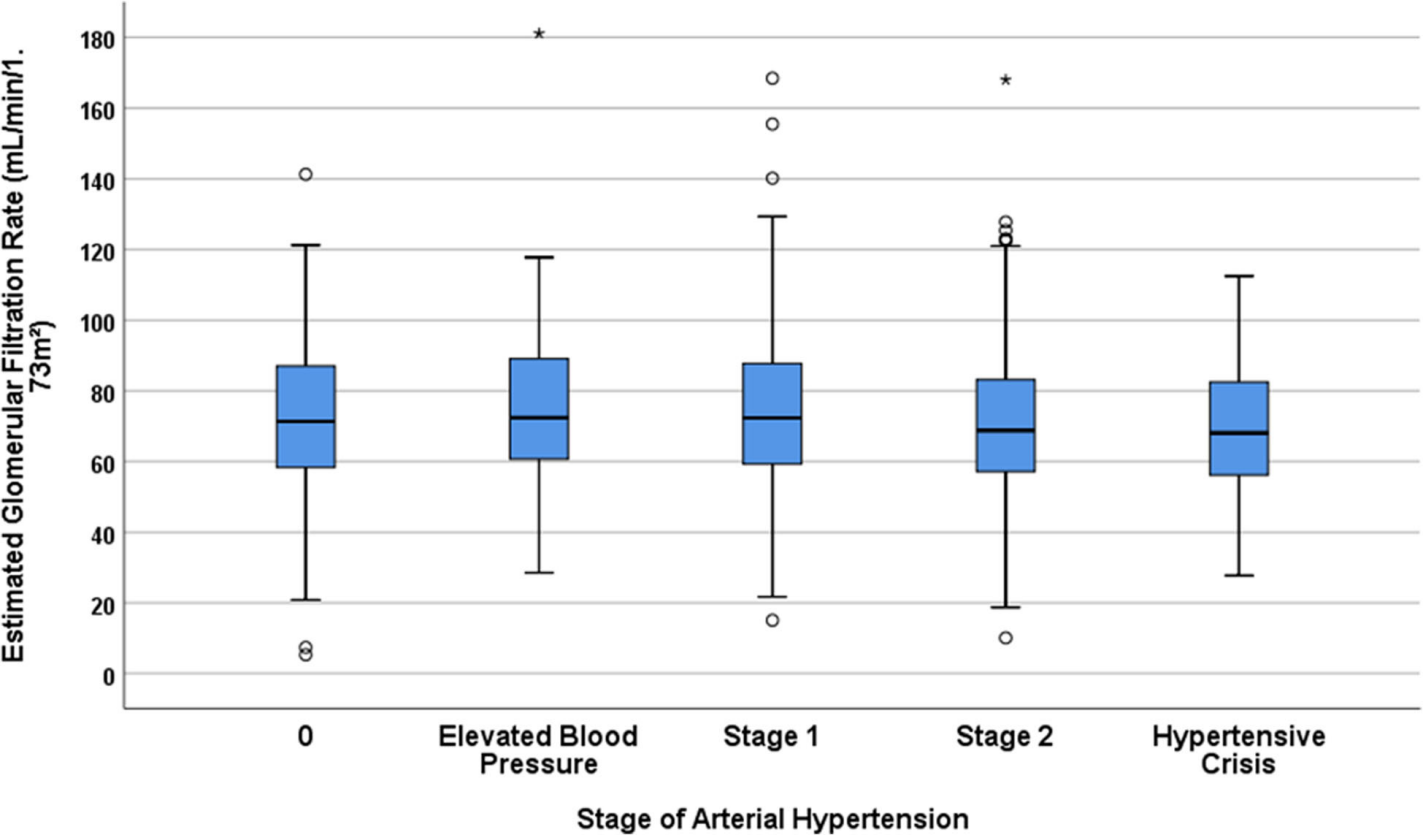
Box plot graph showing the distribution of the estimated glomerular filtration rate stratified by the arterial hypertension stages in the Ural Eye and Medical Study

**Fig. 5:**
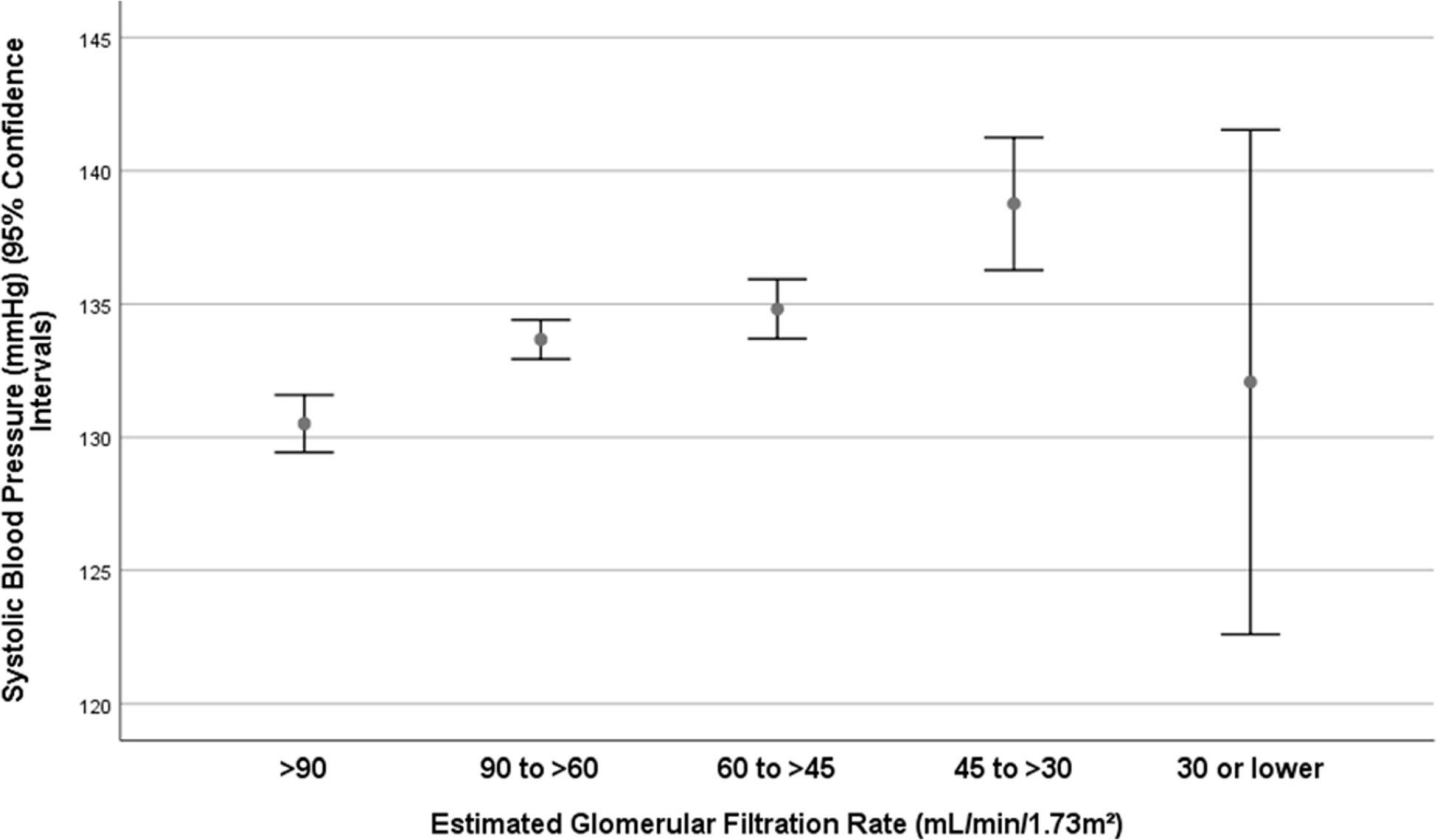
Graph showing the distribution of the systolic blood pressure stratified by the chronic kidney disease stage in the Ural Eye and Medical Study

With increasing stage of arterial hypertension, the eGFR decreased significantly (*P* = 0.04) (Fig. 4). It hold even more true when patients with a CKD stage 4+ were excluded (*P* = 0.02). Correspondingly, systolic blood pressure increased significantly with lower eGFR (*P* < 0.001), although it dropped from CKD stage 3b to stage 4+ (Fig. 5).

Due the relatively strong association between a higher prevalence of CKD stage 3+ and older age (OR: 1.06; 95%CI: 1.06, 1.07; *P* < 0.001), and since many of the independent variables were also associated with age, we conducted the following analysis of associations between the prevalence of CKD stage 3a + after adjusting for age. A higher prevalence of CKD stage 3+ was then significantly correlated with female sex (*P* < 0.001), rural region of habitation (*P* < 0.001), and other parameters (Table 1). In the multivariable analysis, we dropped parameters such as waist-hip ratio due to collinearity, and due to lack of significance, parameters such as mean arterial blood pressure (*P* = 0.89), and others (Table 1). In the final model, a higher prevalence of CKD (stage 3a+) was associated with older age (*P* < 0.001), female sex (*P* < 0.001), rural region of habitation (*P* = 0.001), higher body mass index (*P* = 0.03), lower prevalence of house ownership (*P* = 0.02), higher prevalence of mostly sitting or standing during work (*P* < 0.001), higher serum concentration of triglycerides (*P* < 0.001) and blood urea nitrogen (*P* < 0.001), lower serum concentration of hemoglobin (*P* = 0.03), lower prevalence of diabetes mellitus (*P* = 0.004), and lower prevalence of chronic obstructive pulmonary disease (*P* < 0.001) (Table 2). If the blood urea nitrogen parameter was dropped, all other variables remained to be significantly associated with the prevalence of CKD stage 3a+. If the parameters of systolic blood pressure (*P* = 0.46), diastolic blood pressure (*P* = 0.48) and stage of arterial hypertension (*P* = 0.64) were independently added to the model, there were not statistically significantly associated with the prevalence of CKD stage 3 + .

**Table 1:** Logistic regression analysis of the associations between the prevalence of a chronic kidney disease stage 3a + (estimated glomerular filtration rate < 60 mL/min/1.73m^2^) and systemic parameters after adjusting for age in the Ural Eye and Medical Study

Parameter	Total Study Population	Estimated Glomerular Filtration Rate ≥ 60 mL/min/1.73m^2^ (*n* = 4167)	Estimated Glomerular Filtration Rate < 60 mL/min/1.73m^2^ (*n* = 1674)	*P*-Value	Odds ratio	95% Confidence Intervals
Gender: Women / Men (*n* = 5841)	3291 (56.3%) / 2550 (43.7%)	2121 (50.9%) / 2046 (49.1%)	1170 (69.9%) / 504 (30.1%)	< 0.001	2.20	1.94, 2.49
Rural / urban region of habitation (n = 5841)	3385 (42.0%) / 2456 (42.0%)	2378 (57.1%) / 1789 (42.9%)	1007 (60.2%) / 667 (39.8%)	< 0.001	1.56	1.38, 1.77
Family status: Married versus any other status (n = 5841)	4269 (73.1%) / 1570 (26.9%)	3181 (76.3%)	1088 (65.0%)	< 0.001	0.77	0.68, 0.88
Family type^a^: Joint (three generations) / nuclear (two generations) / single / family of 2 people (*n* = 5821)	1523 (26.2%) / 2479 (42.6%) / 322 (5.5%) / 1497 (25.7%)	1077 (25.8%) / 1812 (43.6%) / 200 (4.8%) / 1071 (25.7%)	446 (26.9%) / 667 (40.2%) / 122 (7.3%) / 426 (25.6%)	0.25	1.08	0.95, 1.24
Ethnicity: Russian / any other ethnicity (n = 5841)	1180 (22.0%)	845 (22.2%)	335 (21.5%)	0.03	0.84	0.73, 0.98
Body mass index (kg/m^2^) (n = 5841)	27.9 ± 5.0	27.8 ± 5.0	28.3 ± 4.9	< 0.001	1.02	1.01, 1.03
Waist-hip-circumference-ratio (*n* = 5837)	0.91 ± 0.09	0.91 ± 0.09	0.91 ± 0.09	0.01	0.42	0.21, 0.82
Socioeconomic parameters						
Level of education (*n* = 5839)^b^	5.6 ± 1.4	5.6 ± 1.3	5.3 ± 1.5	0.92	1.00	0.96, 1.04
House ownership (n = 5841)	5581 (95.5%)	3963 (95.1%)	1618 (96.7%)	< 0.001	0.74	0.63,0.87
Socioeconomic Score (*n* = 5836)^c^	9.7 ± 1.8	9.8 ± 1.8	9.4 ± 1.9	0.23	0.98	0.95, 1.01
Physical activity						
How long is your usual work day? (Minutes) (*n* = 4324)	463 ± 263	488 ± 271	394 ± 227	< 0.001	0.999	0.999, 1.000
Does your work involve mostly sitting or standing with less than 10 min of walking at a time? (*n* = 5066)	3456 (68.2%)	493 (68.0%)	963 (68.9%)	< 0.001	1.42	1.23, 1.64
History of diseases						
History of angina pectoris (n = 5840)	533 (9.1%)	386 (9.3%)	147 (8.8%)	0.56	1.06	0.87, 1.31
History of asthma (n = 5840)	162 (2.8%)	115 (2.8%)	47 (2.8%)	0.23	0.80	0.56, 1.15
History of arthritis (n = 5840)	1618 (27.7%)	1102 (26.5%)	516 (30.8%)	0.74	0.98	0.86, 1.12
History of previous bone fractures (*n* = 5355)	1636 (30.6%)	1192 (31.4%)	444 (28.5%)	0.001	0.80	0.70, 0.92
History of low back pain (*n* = 5355)	2893 (54.0%)	2052 (54.0%)	841 (54.0%)	0.73	1.02	0.90, 1.16
History of thoracic spine pain (n = 5355)	1255 (23.4%)	854 (22.5%)	401 (25.8%)	0.007	1.22	1.06, 1.40
History of neck pain (n = 5355)	1559 (29.1%)	1111 (29.3%)	448 (28.8%)	0.97	1.00	0.87, 1.14
History of headache (n = 5355)	2530 (47.2%)	1752 (46.1%)	778 (50.0%)	0.007	1.19	1.05, 1.34
History of cancer (n = 5355)	174 (3.0%)	109 (2.6%)	65 (3.9%)	0.51	1.12	0.81, 1.54
History of cardiovascular disorders including stroke (n = 5840)	1456 (27.2%)	954 (25.1%)	502 (32.2%)	0.53	1.04	0.91, 1.20
History of dementia (n = 5355)	37 (0.7%)	22 (0.6%)	15 (1.0%)	0.71	0.88	0.45, 1.74
History of diarrhea (n = 5355)	27 (0.5%)	20 (0.5%)	7 (0.4%)	0.73	0.86	0.35, 2.09
History of iron-deficiency anemia (n = 5355)	303 (5.%)	216 (5.7%)	87 (5.6%)	0.29	1.16	0.89, 1.51
History of low blood pressure and hospital admittance (*n* = 5813)	214 (3.7%)	148 (3.6%)	66 (3.9%)	0.64	0.93	0.68, 1.28
History of osteoarthritis (n = 5355)	981 (18.3%)	687 (18.1%)	294 (18.9%)	0.58	0.96	0.82, 1.12
History of skin disease (n = 5355)	280 (5.2%)	193 (5.1%)	87 (5.6%)	0.56	1.08	0.83, 1.42
History of thyreopathy (n = 5840)	604 (10.3%)	410 (9.8%)	194 (11.6%)	0.18	1.14	0.94, 1.37
History of falls (n = 5836)	1099 (18.8%)	793 (19.1%)	306 (18.3%)	0.02	0.84	0.72, 0.98
History of unconsciousness (n = 5840)	486 (8.3%)	334 (8.0%)	152 (9.1%)	0.80	1.03	0.83, 1.27
Blood concentrations of:						
Alanine aminotransferase (IU/L) (*n* = 5835)	21.2. ± 12.1	21.3 ± 12.6	20.9 ± 10.8	0.14	1.00	1.00, 1.00
Aspartate aminotransferase (IU/L) (n = 5838)	20.8 ± 11.0	20.8 ± 11.3	20.6 ± 10.1	0.24	1.00	0.99, 1.00
Bilirubin, total (μmol/L) (n = 5841)	14.9 ± 11.2	14.9 ± 11.1	15.0 ± 11.5	0.59	1.00	1.00, 1.01
High-density lipoproteins (mmol/L) (*n* = 5315)	2.32 ± 0.89	2.32 ± 0.87	2.30 ± 0.95	0.46	0.97	0.91, 1.04
Low-density lipoproteins (mmol/L) (*n* = 5316)	2.13 ± 1.20	2.10 ± 1.21	2.20 ± 1.17	0.01	1.07	1.02, 1.12
Cholesterol (mmol/L) (n = 5841)	5.79 ± 1.69	5.72 ± 1.74	5.96 ± 1.53	< 0.001	1.08	1.04, 1.12
Triglycerides (mmol/L) (*n* = 5320)	1.41 ± 0.75	1.38 ± 0.74	1.48 ± 0.76	< 0.001	1.19	1.10, 1.29
Rheumatoid factor (IU/mL) (n = 5839)	0.12 ± 0.94	0.10 ± 0.82	0.17 ± 1.20	0.67	1.01	0.96, 1.07
Erythrocyte sedimentation rate (mm / hour) (n = 5838)	14.2 ± 11.3	13.4 ± 10.9	16.1 ± 12.1	< 0.001	1.01	1.01, 1.02
Glucose (mmol/L) (n = 5839)	5.03 ± 1.67	5.02 ± 1.72	5.06 ± 1.56	0.06	0.97	0.93, 1.00
Creatinine (μmol/L) (n = 5841)	89.9 ± 25.0	80.9 ± 16.5	112.1 ± 28.4			
Blood urea nitrogen (mmol/L) (n = 5841)	5.11 ± 1.46	4.89 ± 1.25	5.66 ± 1.85	< 0.001	1.34	1.28, 1.40
Residual nitrogen (g/L) (n = 5840)	0.25 ± 0.07	0.25 ± 0.08	0.27 ± 0.06	< 0.001	316	86, 1194
Total protein (g/L) (n = 5841)	76.0 ± 6.3	76.0 ± 6.3	76.0 ± 6.5	0.27	1.01	1.00, 1.02
International normalized ratio (INR) (n = 5837)	1.06 ± 0.14	1.06 ± 0.15	1.06 ± 0.14	0.48	1.16	0.77, 1.75
Prothrombin time (%) (n = 5839)	96.0 ± 10.3	96.0 ± 10.1	96.1 ± 10.6	0.34	1.00	0.99, 1.00
Hemoglobin (g/L) (n = 5840)	142.6 ± 14.8	143.7 ± 14.7	139.9 ± 14.6	< 0.001	0.99	0.98, 0.99
Leukocytes (10^9^ cells / L) (*n* = 5840)	5.12 ± 1.43	5.12 ± 1.41	5.12 ± 1.48	0.58	0.99	0.95, 1.03
Clinical Characteristics						
Prevalence of diabetes mellitus (n = 5841)	680 (11.6%)	468 (11.2%)	212 (12.7%)	0.07	0.85	0.71, 1.01
Blood pressure, systolic (mmHg) (*n* = 5833)	133.6 ± 20.5	132.8 ± 20.1	135.5 ± 21.2	0.05	0.997	0.994, 1.000
Blood pressure, diastolic (mmHg) (n = 5833)	82.0 ± 10.4	82.1 ± 10.4	81.7 ± 10.5	0.04	0.994	0.998, 1.000
Blood pressure, mean (mmHg) (n = 5833)	99.2 ± 12.5	99.0 ± 12.5	99.6 ± 12.6	0.03	0.995	0.990, 0.999
Prevalence of arterial hypertension (n = 5833)	4933 (84.5%)	3486 (83.7%)	1447 (86.5%)	0.31	1.07	0.94, 1.20
Prevalence of chronic obstructive pulmonary disease (*n* = 5350)	367 (6.9%)	290 (7.6%)	77 (4.9%)	< 0.001	0.59	0.45, 0.78
Diet						
Vegetarian diet / mixed diet (n = 5841)	10 (0.2%) / 5831 (99.8%)	8 (0.2%) / 4159 (99.8%)	2 (0.1%) / 1672 (99.9%)	0.98	1.02	0.21, 4.97
Number of meals per day (n = 5836)	3.63 ± 0.79	3.62 ± 0.80	3.66 ± 0.78	0.12	1.06	0.99, 1.14
In a week how many days do you eat fruits? (*n* = 5799)	5.36 ± 1.98	5.36 ± 1.98	5.37 ± 1.98	0.12	1.02	0.99, 1.06
In a week how many days do you eat vegetables? (*n* = 5829)	6.26 ± 1.42	6.27 ± 1.42	6.25 ± 1.42	0.41	1.02	0.98, 1.06
Type of oil used for cooking: vegetable oil / non-vegetable oil (*n* = 4585)	4435 (96.7%) / 150 (3.3%)	3059 (96.3%) / 117 (3.7%)	1376 (97.7%) / 33 (2.3%)	0.10	0.82	0.64, 1.04
Foods Containing Whole Grain (*n* = 5352) (No/Yes)	1089 (20.3%) / 4263 (79.7%)	775 (20.4%) / 3022 (79.6%)	314 (20.2%) / 1241 (79.8%)	0.21	1.10	0.95, 1.29
Salt consumed per day (g) (n = 5316)	4.27 ± 2.37	4.26 ± 2.32	4.29 ± 2.50	0.49	1.01	0.98, 1.04
Degree of processing of meat (weak / medium / well done) (n = 5350)	118 (2.2%) / 1906 (35.6%) / 3326 (62.2%)	94 (2.5%) / 1355 (35.7%) / 2346 (61.8%)	24 (1.5%) / 551 (35.4%) / 980 (63.0%)	0.08	1.11	0.99, 1.24
Smoking						
Do you currently smoke any tobacco products? (yes) (*n* = 5834)	733 (12.6%)	629 (15.1%)	104 (6.2%)	< 0.001	0.49	0.40, 0.62
Do you smoke daily? (yes / no) (n = 5841)	707 (12.1%)	608 (14.6%)	99 (5.9%)	< 0.001	0.48	0.39, 0.61
Package years (package = 20 cigarettes) (*n* = 5800)	4.1 ± 12.7	4.8 ± 13.6	2.2 ± 9.9	< 0.001	0.99	0.98, 0.99
Alcohol Consumption						
Alcohol consumed such as beer, whisky, rum, gin brandy or other local products? (yes / no) (n = 5838)	1238 (21.2%)	940 (22.6%)	298 (17.8%)	0.43	0.94	0.81, 1.10
How many alcoholic drinks (in mL) do you have on a typical day when you are drinking) (*n* = 1221)	194 ± 154	208 ± 158	152 ± 132	< 0.001	0.997	0.996, 0.998
Depression and State-Trait Anxiety Inventory (STAI)						
Depression score (*n* = 5838)	1.18 ± 3.75	1.03 ± 3.70	1.57 ± 3.83	0.009	1.02	1.01, 1.04
Anxiety score (n = 5835)	−0.66 ± 3.55	−0.83 ± 3.53	−0.24 ± 3.55	< 0.001	1.03	1.02, 1.05
Dynamometry						
Manual dynamometry, right hand (dekaNewton) (*n* = 5348)	30.5 ± 11.7	32.2 ± 11.7	26.4 ± 10.7	< 0.001	0.97	0.96, 0.98
Manual dynamometry, left (dekaNewton) (*n* = 5342)	26.9 ± 11.3	28.6 ± 11.4	23.0 ± 10.1	< 0.001	0.97	0.96, 0.98

*P*-values and confidence intervals were not corrected for multiple comparisons

The averages presented in the table are calculated from the numbered ranks assigned to each level

^a^ Joint Family: All non-joint family types (nuclear, single and family) were grouped into one category of “Non-Joint Family” and compared with “Joint Family” (Reference)

^b^ The level of education was categorized into the stages of category 1 (“illiteracy”: no reading ability at all; *n* = 17 (0.3%)), category 2 (“passing of the 5th class”; *n* = 102 (1.7%)), category 3 (“passing of the 8th class”; *n* = 589 (10.1%)), category 4 (“passing of the 10th class”; *n* = 651 (11.1%)), category 5 (“passing of the 11th class”; *n* = 777 (13.3%)), category 6 (“graduation”; *n* = 2029 (34.7%)), and category 7 (“post-graduation and specialized secondary education”; n = 1674 (28.7%))

^c^ The socioeconomic score was calculated as the sum of the level of education (1: “illiteracy”; 2: “passing of the 5th class”; 3: “passing of the 8th class”; 4: “passing of the 10th class”; 5: “passing of the 11th class”; 6: “graduation”; 7: “post-graduation and specialized secondary education”), the level of self-reported income (1: “below the poverty line” (*n* = 1309; 22.4%); 2: “average” (*n* = 4269; 73.1%); 3: “above the average” (*n* = 253; 4.3%); 4: “high” (*n* = 7;0.1%)), ownership of a house (0: no (260; 4.5%); 1: yes (*n* = 5581; 95.5%), and the ownership of a television set (0:no (*n* = 44; 0.8%); 1: yes (*n* = 5797; 99.2%)). A higher score indicates a better socio-economic status

**Table 2:** Associations (multivariable logistic regression analysis) of the prevalence of chronic kidney disease stage 3a + (estimated glomerular filtration rate < 60 mL/min/1.73m^2^) and systemic parameters in the Ural Eye and Medical Study (*n* = 4655)

Parameter	*P*-Value	Odds Ratio	95% Confidence Interval
Age (Years)	< 0.001	1.06	1.05, 1.07
Gender (Women / Men)	< 0.001	2.29	1.94, 2.69
Rural region of habitation	0.001	1.29	1.11, 1.50
Body mass index (kg/m^2^)	0.03	1.02	1.002, 1.03
House ownership	0.02	0.57	0.35, 0.92
Does your work involve mostly sitting or standing with less than 10 min of walking at a time?	< 0.001	1.40	1.20, 1.64
Serum concentration of blood urea nitrogen concentration (mmol/L)	< 0.001	1.33	1.27, 1.40
Serum concentration of hemoglobin (g/L)	0.03	0.99	0.99, 0.999
Serum concentration of triglycerides (mmol/L)	< 0.001	1.23	1.12, 1.35
Diabetes Mellitus	0.004	0.71	0.56, 0.90
Chronic obstructive pulmonary disease	< 0.001	0.57	0.42, 0.78

The multivariable logistic regression analysis included as independent variables all those parameters that were associated (*P* < 0.10) with the CKD stage 3a + prevalence in the previous analysis after adjusting for age (age, gender, region of habitation, family status, Russian ethnicity, body mass index, waist-hip circumference ratio, house ownership, length of usual working day, work performed mostly in sitting position, history of previous bone fractures, thoracic spine pain, headache and previous falls, serum concentrations of lipoproteins, cholesterol, triglycerides, glucose, blood urea nitrogen, residual nitrogen and hemoglobin, erythrocyte sedimentation rate, systolic, diastolic and mean blood pressure, prevalence of diabetes mellitus and chronic obstructive pulmonary disease, degree of process meat, current smoking, daily smoking, smoking package years, number of alcoholic drinks (in mL) on a typical day with alcohol drinking, depression score, anxiety score, and left and right manual dynamometry). We then dropped parameters due to collinearity (such as waist-hip ratio, left dynamometry, systolic and diastolic blood pressure, prevalence of arterial hypertension, serum concentration of glucose) and due to lack of significance (such as mean arterial blood pressure (*P* = 0.89), and others)

## Discussion

In our population-based study on a population from Russia with a typical ethnical composition, the prevalences of CKD stage 3a, 3b and 4 were 23.1, 5.0 and 0.5%, respectively. The prevalence of CKD stage 3a or higher increased from 11.1% in the age group of 40–44 years to 21.0% in the age group of 50–54 years, and to 56.8% (95%CI:52.8,60.8) in the age group of 75+ years. In univariate analysis, the prevalence of CKD stage 3a + increased with higher systolic blood pressure (*P* < 0.001). Higher prevalence of CKD stage 3+ was not significantly associated with a higher prevalence of diabetes mellitus (Fig. 3). In multivariable analysis, higher prevalence of CKD stage 3a + was associated with older age, female sex, rural region of habitation, higher body mass index, lower prevalence of house ownership, higher prevalence of mostly sitting or standing during work, higher serum concentration of triglycerides and blood urea nitrogen and lower serum concentration of hemoglobin, and lower prevalence of diabetes mellitus and of chronic obstructive pulmonary disease.

The findings obtained in our study on the prevalence of CKD in Russia can hardly directly be compared with other investigations from Russia due to the scarcity of such studies in the country. In the study by van Pottelbergh and colleagues on the prevalence of CKD in 611 individuals aged 65+ years) in a district of St. Petersburg, the prevalence of CKD stage III-V for men ranged between 11 and 15% and for females between 14 and 29% [6]. Including only individuals with an age of 65+ year from our study population revealed a prevalence of CKD stage 3+ of 37.9% (95%CI: 34.2, 41.6) for men and of 49.4% (95%CI: 46.5, 52.4) for women. These figures were higher than those reported by van Pottelbergh and associates. In a study by Dobronravov and colleagues performed in the towns of Veliky Novgorod and Syktyvkar in the North-West region of the Russian Federation from 1998 to 1999, 490 patients with CKD of stage IV+ were found in a total population of 1,840,000, revealing a prevalence of 266 per million or 0.03% [4]. In an investigation conducted by Smirnov and coworkers in the Tyva Republic in Southern Siberia in the period from 2003 to 2004, the prevalence of CKD stage IV+ was 493 patients per million or 0.0493% [5]. These figures were by a factor of 10 or 18, respectively, lower than the prevalence of CKD4+ in our study population (32/5841 or 0.55%). One of the reasons for the discrepancies between the studies may be differences in the study period and associated differences in the medical infrastructure. The less developed the medical infrastructure was, the lower might have been the life expectancy of patients with CKD, and the medical infrastructure was less developed in 1998/1999 as compared to our study period of 2015 to 2017. In addition, it depended on the presence of diabetes mellitus and diastolic blood pressure.

Compared with other countries, the prevalence of CKD stage 3+ was higher in our study population from Russia than in a worldwide meta-analysis in which the prevalence of CKD stage 3a + b was 7.6% (95%CI: 6.4, 8.9) [22]. The worldwide figures for the prevalence of CKD stage 4 (0.4%; 95%: 0.3, 0.5) and CKD stage 5 (0.1%; 95%CI: 0.1, 0.1) were similar to the one in our study population [22]. Previous studies have shown a dependence of the prevalence of CKD on geography and on the socio-demographic index, with a higher CKD prevalence in regions such as Europe, USA, Canada and Australia [22]. Although Russia does not belong to the high-income countries, its medical infrastructure has now been relatively well developed. To cite an example, the awareness rate of diabetes mellitus in the Ural Eye and Medical Study was 500/687 or 72.8% (95%CI: 69.0, 76.0), a figure fully comparable with the general diabetes awareness rate of 74.8% in the U.S.A., but considerably higher than the values reported for Hispanics/Latinos living in the U.S.A. with an awareness rate of 58.7% [23–26].

The relatively high prevalence of CKD in the present study population may therefore be associated with the medical infrastructure in the study region, leading to an increasing survival time of patients with CKD and thus a higher percentage of this group in the total population. Other reasons for discrepancies between various studies in the prevalence of CKD may be due to differences in other parameters such diet, environmental factors and others.

The associations between a higher prevalence of CKD and systemic factors such as higher age and female gender are in agreement with the results of previous studies from other countries. In the worldwide meta-analysis, two-thirds of the 100 studies included into the analysis found a higher CKD prevalence in women than in men [22, 26–28]. The reasons for this gender-specific difference have remained elusive so far. The lower muscle mass in women as compared to men with the muscle mass being a major factor of the serum creatinine concentration has been taken into account in the calculation of the eGFR values [12–14, 27–29].

Interestingly, a higher prevalence of CKD was not associated with a higher prevalence of diabetes mellitus in the multivariable analysis (Table 2), while in the univariate analysis, the prevalence of diabetes mellitus increased in the CKD stage 4+ (Fig. 3). Reason for the lack of a clear association between diabetes mellitus and CKD in the study population may be that patients with diabetes were relatively early detected (according to the high awareness rate of 72.8%), so that diabetes-related late sequelae such as CKD might have occurred in a relatively low frequency [23]. In that context, it should be taken into account that early diabetic nephropathy is characterized by albuminuria, which was not measured in our study.

When results of our study are discussed, its limitations should be considered. First, the study depended on a single measurement of the serum creatinine concentration, which however varies within individuals with changes up to 21% within a 2-week period [30]. Correspondingly, a population-based study from Morocco reported that up to 30% of the individuals initially classified as CKD 3a showed higher eGFR values 12 months later and were thus no longer considered having a CKD [31]. Second, when comparing results between different studies and countries, differences in the methods applied and differences in the formulas used to calculate the eGFR values have to be taken into account. Third, when the results of investigations on different study populations are compared with each other, differences in the composition of the study populations with respect to the factors associated with the prevalence of CKD should be considered. These factors include the parameters of age, gender, inclusion and exclusion criteria, study period and quality of the medical infrastructure. In particular the latter factor may influence the difference in the prevalence of CKD between high-income countries and low-income countries where patients with CKD have a shortened remaining life expectancy so that their proportion on the general population diminishes. Fourth, since we did not examine the prevalence of proteinuria in this population-based investigation, the diagnosis of CKD could be based only on the eGFR value.

## Conclusions

In conclusion, in this population from Russia with an age of 40+ years, the prevalence of CKD stage 3+ (28.7%) was relatively high as compared to populations from other countries. Factors associated with the prevalence of CKD were older age, female sex, rural region of habitation, higher body mass index, higher prevalence of more sedentary lifestyle, and a lower socioeconomic background.

## Data Availability

Available on request from the corresponding author.

## Abbreviations

GBD: Global burden of diseases, injuries and risk factors
CKD: Chronic kidney disease
CI: Confidence intervals
YLL: Year of live lost
GFR: Glomerular filtration rate
DALY: Disability-adjusted life years
GATHER: Guidelines for Accurate and Transparent Health Estimates Reporting
BP: Blood pressure
SBP: Systolic blood pressure
DBP: Diastolic blood pressure
eGFR: Estimated glomerular filtration rate
CKD-EPI: CKD Epidemiology Collaboration
SPPS: Statistical package for social science
OR: Odds ratio
Fig: Figure

## References

[1] GBD 2017 Causes of Death Collaborators (2018). Global, regional, and national age-sex-specific mortality for 282 causes of death in 195 countries and territories, 1980–2017: a systematic analysis for the Global Burden of Disease Study 2017. Lancet.

[2] GBD 2017 Risk Factor Collaborators (2018). Global, regional, and national comparative risk assessment of 84 behavioural, environmental and occupational, and metabolic risks or clusters of risks for 195 countries and territories, 1990–2017: a systematic analysis for the Global Burden of Disease Study 2017. Lancet.

[3] GBD 2016 Russia Collaborators (2018). The burden of disease in Russia from 1980 to 2016: a systematic analysis for the Global Burden of Disease Study 2016. Lancet.

[4] Dobronravov VA, Smirnov AV, Dragunov SV, Zver'kov RV, Evdokimova TV, Butrimova SS, Grigorshchuk VI (2004). Epidemiology of chronic renal disease in the north-west of Russia: setting-up the register. Ter Arkh.

[5] Smirnov AV, Dobronravov VA, Bodur-Oorzhak AS, Zver'kov RV, Larionova VI, Glazkov PB, Bogdanova MA, Mnuskina MM, Kaiukov IG, Sanchi MN (2005). Epidemiology and risk factors of chronic renal diseases: a regional level of the problem. Ter Arkh.

[6] Van Pottelbergh C, Gurina N, Degryse J, Frolova E (2011). Prevalence of impaired renal function in the elderly in the St. Petersburg District: results of the crystal study. Adv Gerontol.

[7] Gurina NA, Frolova EV, Degryse JM (2011). A roadmap of aging in Russia: the prevalence of frailty in community-dwelling older adults in the St. Petersburg district--the “crystal” study. J Am Geriatr Soc..

[8] Rutkowski B (2000). Changing pattern of end-stage renal disease in central and eastern Europe. Nephrol Dial Transplant.

[9] Sedov KR, Vereshchagina TD (1993). The epidemiology of the most prevalent kidney diseases in the nationalities of the north. Urol Nefrol (Mosk).

[10] Ryabov SI, Stavskaya VV (1983). Epidemiology of chronic renal diseases. Int Urol Nephrol.

[11] Bikbov M, Fayzrakhmanov RR, Kazakbaeva G, Jonas JB (2018). Ural eye and medical study: description of study design and methodology. Ophthalmic Epidemiol.

[12] Bikbov MM, Fayzrakhmanov RR, Kazakbaeva GM, Zainullin RM, Salavatova VF, Gilmanshin TR, Arslangareeva II, Nikitin NA, Panda-Jonas S, Mukhamadieva SR, Yakupova DF, Khikmatullin RI, Aminev SK, Nuriev IF, Zaynetdinov AF, Uzianbaeva YV, Jonas JB (2018). Frequency and associated factors of bone fractures in Russians: the Ural eye and medical study. Sci Rep.

[13] Bikbov MM, Fayzrakhmanov RR, Kazakbaeva GM, Zainullin RM, Salavatova VF, Gilmanshin TR, Arslangareeva II, Nikitin NA, Panda-Jonas S, Mukhamadieva SR, Yakupova DF, Khikmatullin RI, Aminev SK, Nuriev IF, Zaynetdinov AF, Uzianbaeva YV, Jonas JB (2019). Self-reported hearing loss in Russians: the population-based Ural eye and medical study. BMJ Open.

[14] Stevens GA, Alkema L, Black RE, Boerma JT, Collins GS, Ezzati M, Grove JT, Hogan DR, Hogan MC, Horton R, Lawn JE, Marušić A, Mathers CD, Murray CJ, Rudan I, Salomon JA, Simpson PJ, Vos T, Welch V, (The GATHER working group) (2016). Guidelines for accurate and transparent health estimates reporting: the GATHER statement. Lancet..

[15] Whelton PK, Carey RM, Aronow WS, Casey DE, Collins KJ, Dennison Himmelfarb C, DePalma SM, Gidding S, Jamerson KA, Jones DW, MacLaughlin EJ, Muntner P, Ovbiagele B, Smith SC, Spencer CC, Stafford RS, Taler SJ, Thomas RJ, Williams KA, Williamson JD, Wright JT (2018). 2017 ACC/AHA/AAPA/ABC/ACPM/AGS/APhA/ASH/ASPC/NMA/PCNA guideline for the prevention, detection, evaluation, and Management of High Blood Pressure in adults: executive summary: a report of the American College of Cardiology/American Heart Association task force on clinical practice guidelines. Hypertension..

[16] Whelton PK, Carey RM, Aronow WS, Casey DE, Collins KJ, Dennison Himmelfarb C, DePalma SM, Gidding S, Jamerson KA, Jones DW, MacLaughlin EJ, Muntner P, Ovbiagele B, Smith SC, Spencer CC, Stafford RS, Taler SJ, Thomas RJ, Williams KA, Williamson JD, Wright JT (2018). 2017 ACC/AHA/AAPA/ABC/ACPM/AGS/APhA/ASH/ASPC/NMA/PCNA guideline for the prevention, detection, evaluation, and Management of High Blood Pressure in adults: a report of the American College of Cardiology/American Heart Association task force on clinical practice guidelines. J Am Coll Cardiol.

[17] Levey AS, Stevens LA, Schmid CH, Zhang YL, Castro AF, Feldman HI, Kusek JW, Eggers P, Van Lente F, Greene T, Coresh J, CKD-EPI (Chronic Kidney Disease Epidemiology Collaboration) (2009). A new equation to estimate glomerular filtration rate. Ann Intern Med.

[18] Hsu CY, ACP Journal Club (2012). CKD-EPI eGFR categories were better than MDRD categories for predicting mortality in a range of populations. Ann Intern Med.

[19] Matsushita K, Mahmoodi BK, Woodward M, Emberson JR, Jafar TH, Jee SH, Polkinghorne KR, Shankar A, Smith DH, Tonelli M, Warnock DG, Wen CP, Coresh J, Gansevoort RT, Hemmelgarn BR, Levey AS (2012). Chronic Kidney Disease Prognosis Consortium Comparison of risk prediction using the CKD-EPI equation and the MDRD study equation for estimated glomerular filtration rate. JAMA.

[20] Parsh J, Seth M, Aronow H, Dixon S, Heung M, Mehran R, Gurm HS (2015). Choice of estimated glomerular filtration rate equation impacts drug-dosing recommendations and risk stratification in patients with chronic kidney disease undergoing percutaneous coronary interventions. J Am Coll Cardiol.

[21] https://en.wikipedia.org/wiki/Demographics_of_Russia. Assessed 4.2.2019.

[22] Hill NR, Fatoba ST, Oke JL, Hirst JA, O'Callaghan CA, Lasserson DS, Hobbs FD (2016). Global prevalence of chronic kidney disease - a systematic review and meta-analysis. PLoS One.

[23] Bikbov MM, Fayzrakhmanov RR, Kazakbaeva GM, Zainullin RM, Arslangareeva II, Gilmanshin TR, Salavatova VF, Nikitin NA, Mukhamadieva SR, Yakupova DF, Khikmatullin RI, Zaynetdinov AF, Uzianbaeva YV, Aminev SK, Nuriev IF, Jonas JB (2019). Prevalence, awareness and control of diabetes in Russia: the Ural eye and medical study on adults aged 40+ years. PLoS One.

[24] Menke A, Casagrande S, Geiss L, Cowie CC (2015). Prevalence of and trends in diabetes among adults in the United States, 1988-2012. JAMA.

[25] Schneiderman N, Llabre M, Cowie CC, Barnhart J, Carnethon M, Gallo LC, Giachello AL, Heiss G, Kaplan RC, LaVange LM, Teng Y, Villa-Caballero L, Avilés-Santa ML (2014). Prevalence of diabetes among Hispanics/Latinos from diverse backgrounds: the Hispanic community health study/study of Latinos (HCHS/SOL). Diabetes Care.

[26] Ene-Iordache B, Perico N, Bikbov B, Carminati S, Remuzzi A, Perna A, Islam N, Bravo RF, Aleckovic-Halilovic M, Zou H, Zhang L, Gouda Z, Tchokhonelidze I, Abraham G, Mahdavi-Mazdeh M, Gallieni M, Codreanu I, Togtokh A, Sharma SK, Koirala P, Uprety S, Ulasi I, Remuzzi G (2016). Chronic kidney disease and cardiovascular risk in six regions of the world (ISN-KDDC): a cross-sectional study. Lancet Glob Health.

[27] Eriksen BO, Ingebretsen OC (2006). The progression of chronic kidney disease: a 10-year population-based study of the effects of gender and age. Kidney Int.

[28] Carrero JJ (2010). Gender differences in chronic kidney disease: underpinnings and therapeutic implications. Kidney Blood Press Res.

[29] Neugarten J, Acharya A, Silbiger SR (2000). Effect of gender on the progression of nondiabetic renal disease: a meta-analysis. J Am Soc Nephrol.

[30] Coresh J, Astor BC, McQuillan G, Kusek J, Greene T, Van Lente F, Levey AS (2002). Calibration and random variation of the serum creatinine assay as critical elements of using equations to estimate glomerular filtration rate. Am J Kidney Dis.

[31] Benghanem Gharbi M, Elseviers M, Zamd M, Belghiti Alaoui A, Benahadi N, Trabelssi el H, Bayahia R, Ramdani B, De Broe ME (2016). Chronic kidney disease, hypertension, diabetes, and obesity in the adult population of Morocco: how to avoid “over”- and “under”-diagnosis of CKD. Kidney Int.

